# Priapism for 10 days in a patient with SARS-CoV-2 pneumonia: a case report

**DOI:** 10.1093/jscr/rjab020

**Published:** 2021-04-13

**Authors:** Abdulmalik Addar, Omar Al Fraidi, Ahmed Nazer, Naif Althonayan, Yahya Ghazwani

**Affiliations:** 1 Department of Surgery, Division of Urology Surgery, Ministry of National Guard-Health Affairs, King Abdulaziz Medical City, Riyadh, Saudi Arabia; 2 College of Medicine, King Saud Bin Abdulaziz University for Health Sciences, Riyadh, Saudi Arabia; 3 King Abdullah International Medical Research Center, Riyadh, Saudi Arabia

## Abstract

Severe acute respiratory syndrome coronavirus 2 (SARS-CoV-2) has been linked to thromboembolic complications. Priapism has been reported only once in link to SARS-CoV2.

Here we report the second case of priapism in a patient with SARS-CoV2; our case is unique in being that the patient had priapism for 10 days while being hospitalized. We discuss potential causes and possible prevention strategies. The patient was managed by aspiration and Phenylephrine injection and achieved detumescence and reported normal erection at 2 weeks follow-up.

## INTRODUCTION

Severe acute respiratory syndrome coronavirus 2 (SARS-CoV-2) is the strain of coronavirus that causes coronavirus disease 2019 (COVID-19), which started in late 2019 and has become a global pandemic [1]. Clinical presentation, when symptomatic, is usually in the form of pneumonia that may become severe and causes Acute Respiratory Distress Syndrome, other life threating complications from COVID-19 include venous and arterial thromboembolism [1, 2]. Ischemic priapism is caused by veno-occlusion, which can be caused by hyper coagulability and progressive time-dependent ischemia, which eventually leads to smooth muscle necrosis [3].

**Figure 1:**
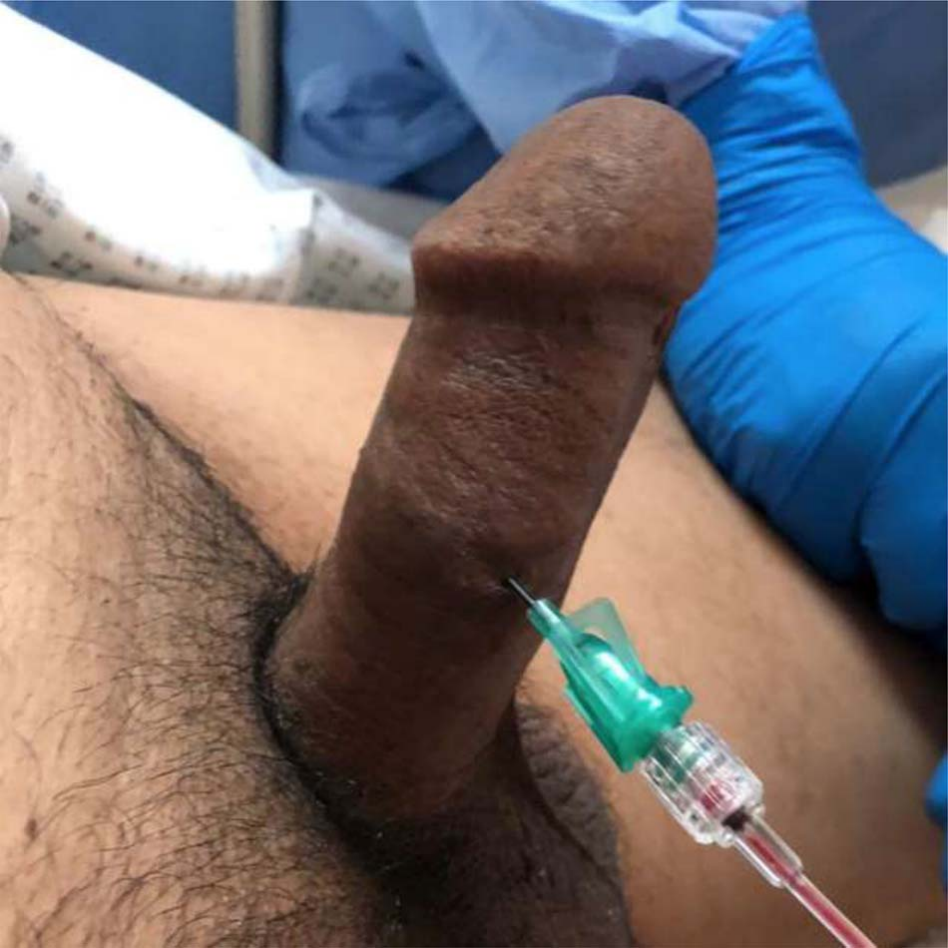
Patients penis prior to starting aspiration and awaiting penile blood gasses.

**Figure 2:**
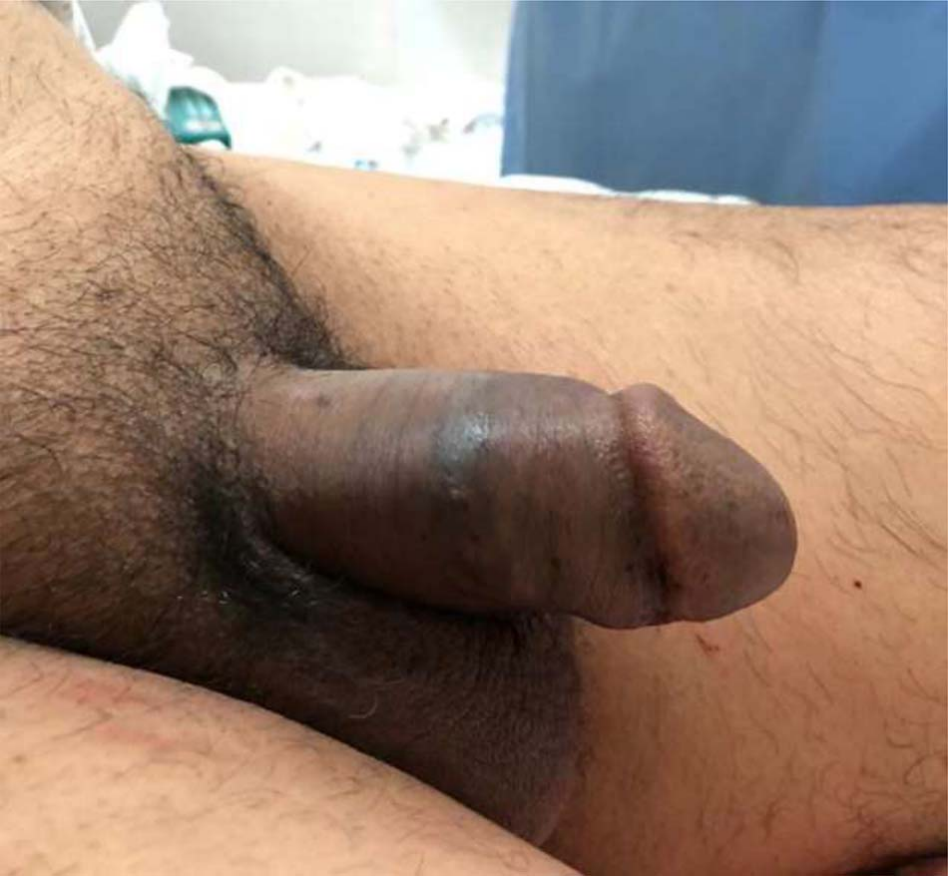
Patients penis after aspiration achieved detumescence.

Here we report a peculiar incident of 10 days priapism in a patient admitted as a case of COVID-19 pneumonia. This is the second case report of priapism in COVID-19 patients and the first with this presentation. We report this case in accordance with the CARE reporting guidelines checklist.

## CASE REPORT

A 62-year-old male known to have hypertension and dyslipidemia, presented to our hospital with shortness of breath, fatigue and dry cough. Chest x-ray for the patient showed patchy infiltrates in the right lung. The patient was admitted under Internal Medicine service as a case of suspected COVID-19 pneumonia, which was later confirmed by polymerase chain reaction (PCR) test and was put on Enoxaparin 40 mg daily, IV fluids and acetaminophen upon admission according to hospital protocol. On day three of admission, patient started to have increased respiratory effort and was requiring oxygen support via high flow nasal cannula. He was then admitted to the intensive care unit (ICU), and his enoxaparin was increased to a therapeutic dose and was started on steroids, broad-spectrum antibiotics and intravenous fluid. A 16 Fr Foley’s catheter was inserted for monitoring urine output and was a routine measure in all ICU patients. After 3 days in the ICU the patient became stable and was transferred to general ward. He noticed that he has a constant painless erection that he thought it is caused by the Foley’s catheter and did not report it to hospital staff. Because of the high volume of COVID-19 patients and as a safety precaution, physicians did most of their rounds via Telemedicine video calls. Therefore, genital exams were only done when there is a complaint made by the patient. After spending 21 days in the hospital the patient was ready for discharge, and while removing the patient’s Foley’s catheter in preparation for discharge, the primary physician noticed the erection and the Urology team were consulted for suspected priapism. The patient reported that he has been having a constant erection for at least 10 days, which he noticed after Foley’s catheter insertion. He denied any history of, pain, trauma, use of erectile dysfunction (ED) medications or treatment. He denied any voiding difficulty after removing the Foleys or prior to his admission. Patient vitals were stable. On examination, patient had a hard erection, no skin changes were noticed, no discoloration or tenderness. Patient labs revealed leukocytosis of 18.6 mostly neutrophils and platelet count of 470. Aspiration with 18 French gauge needle was difficult due to increased blood viscosity however, we managed to withdraw about 10 ml, which had no effect on the erection rigidity (Fig. 1). Blood gases sent from the penis came back with PH = 6.86, pCO2 = 33.3 and, pO2 = 26.9 confirming and diagnosis of ischemic priapism. Phenylephrine 200 μg was then injected into the corpora cavernosa twice 3 minutes apart, and aspiration was continued until detumescence was achieved (Fig. 2). The patient was given prophylactic antibiotics and was discharged 2 days later. On follow-up 2 weeks later via phone call, patient was still under home isolation and reported having nighttime erections with desire, no sexual encounters or ejaculation and, having mild itching at the aspirationsite.

## DISCUSSION

The American Urological Association defines priapism as ‘a persistent penile erection that continues hours beyond, or is unrelated to, sexual stimulation’ [4]. There are three types of priapism (ischemic, non-ischemic and stuttering priapism) [3]. Ischemic priapism is a low flow priapism characterized by decrease venous outflow from the corpora cavernousa. Furthermore, this condition is associated with a hypercoagulability as a cause [3].

SARS-CoV-2 is linked to a hypercoagulability status [5]. The risk of venous thromboembolism with proven SARS-CoV-2 infection has been extensively studied [5, 6]. Klok *et al*. [6] reported a high incidence of venous thromboembolism (VTE) in ICU patients despite being on thromboprophylaxis. The risk of VTE caused by factors such as inflammatory response, activation of the coagulation cascade and by the virus itself. The high number of activated cells by SARS-CoV-2 leads to an increase in blood viscosity [5]. Increase in the neutrophil, lymphocyte and platelet count and neutrophil/lymphocyte ratio appears to be related to disease severity [7]. This increase in blood viscosity may lead to obliteration of the small emissary veins in the subtunical space and subsequent ischemic priapism [3].

Ischemic priapism is a urological emergency [3, 4, 8]. Aspiration of the corporal blood is the initial management followed by irrigation of the corpora then instillation of α-agonists [8]. Furthermore, surgical therapy can be considered if the initial management failed to achieve detumescence [4, 8]. Priapism that lasts more than 48 hours is associated with a high incidence of ED [9].

Lamamri *et al*. [10] reported the first case of priapism in patient with SARS-CoV-2 in a 62-year-old man with priapism for 4 hours upon ICU admission that was treated by corporal aspiration but was not followed up later on. Our patient had priapism for almost 10 days and on 3 weeks follow-up was having normal erections but no sexual activity. In the mentioned case report, they conclude three explanations for priapism among SARS-COV-2, which is similar to what we concluded.

Daily assessment and examination of patients is the cornerstone of medicine. However, during this pandemic many reports revealed the effectiveness of telemedicine in patient assessment.

## CONFLICT OF INTEREST STATEMENT

None declared.

## FUNDING

None.

## References

[1] Machhi J , Herskovitz J, Senan AM, Dutta D, Nath B, Oleynikov MD, et al. The natural history, pathobiology, and clinical manifestations of SARS-CoV-2 infections. J Neuroimmune Pharmacol 2020;3:1–28. 10.1007/s11481-020-09944-5 PMC7373339 32696264

[2] Middeldorp S , Coppens M, van Haaps TF, Foppen M, Vlaar AP, Müller MCA, et al. Incidence of venous thromboembolism in hospitalized patients with COVID-19. J Thromb Haemost 2020;18:1995–2002. 32369666 10.1111/jth.14888 PMC7497052

[3] Muneer A , Ralph D. Guideline of guidelines: priapism. BJU Int 2016;119:204–8. 27860090 10.1111/bju.13717

[4] Montague Drogo K , Jarow J, Broderick G, Dmochowski RR, Heaton J, Lue T, et al. AUA guideline on the management of priapism. J Urol 2003;170:1318–24. 14501756 10.1097/01.ju.0000087608.07371.ca

[5] Abou-Ismail MY , Diamond A, Kapoor S, Arafah Y, Nayak L. The hypercoagulable state in COVID-19: incidence, pathophysiology, and management. Thromb Res 2020;194:101–15. 32788101 10.1016/j.thromres.2020.06.029 PMC7305763

[6] Klok FA , Kruip MJHA, van der Meer NJM, Arbous MS, Gommers DAMPJ, Kant KM, et al. Incidence of thrombotic complications in critically ill ICU patients with COVID-19. Thromb Res 2020;191:145–7. 32291094 10.1016/j.thromres.2020.04.013 PMC7146714

[7] Fan BE , Chong VCL, Chan SSW, Lim GH, Lim KGE, Tan GB, et al. Hematologic parameters in patients with COVID-19 infection. Am J Hematol 2020;95:131–53. 10.1002/ajh.25774 32129508

[8] Salonia A , Eardley I, Giuliano F, Hatzichristou D, Moncada I, Vardi Y, et al. European association of urology guidelines on priapism. Eur Urol. 2014;65:480–9. 24314827 10.1016/j.eururo.2013.11.008

[9] El-Bahnasawy MS , Dawood A, Farouk A. Low-flow priapism: risk factors for erectile dysfunction. BJU Int 2002;89:285–90. 11856112 10.1046/j.1464-4096.2001.01510.x

[10] Lamamri M , Chebbi A, Mamane J, Abbad S, Munuzzolini M, Sarfati F, et al. Priapism in a patient with coronavirus disease 2019 (COVID-19): a case report. Am J Emerg Med 2020;39:P251. 10.1016/j.ajem.2020.06.027 PMC7301054 32732087

